# Determinants of drop-offs in the targeted universal tuberculosis testing care cascade among people with HIV in rural and urban facilities in South Africa

**DOI:** 10.4102/sajhivmed.v27i1.1774

**Published:** 2026-02-12

**Authors:** Katlego Motlhaoleng, Kgomotso Vilakazi-Nhlapo, Kate Shearer, Jonathan Golub, Gary Maartens

**Affiliations:** 1 Department of Medicine, Faculty of Health Sciences, University of Cape Town, Cape Town, South Africa; 2 National Department of Health, Pretoria, South Africa; 3 Center for Tuberculosis Research, Johns Hopkins University, Baltimore, United States; 4 Department of Medicine, Division of Infectious Diseases, Johns Hopkins School of Medicine, Baltimore, United States

**Keywords:** TB screening, targeted universal TB Testing, drop-offs, TB care cascade, people with HIV, TB/HIV integration, differentiated service delivery

## Abstract

**Background:**

Targeted Universal Tuberculosis Testing (TUTT) is a strategy for early tuberculosis (TB) detection among people with HIV (PWH); however, drop-offs at key cascade stages limit its effectiveness.

**Objectives:**

This study examines determinants of drop-offs at three stages: rapid molecular diagnostic test for TB (Xpert) TB treatment initiation, and completion.

**Method:**

We conducted a retrospective analysis of routinely collected data in fiscal year 2022 from PWH on antiretroviral therapy (ART) in rural and urban facilities in KwaZulu-Natal, South Africa. Logistic regression identified determinants of drop-offs.

**Results:**

Among 104 859 PWH, 66.7% were not tested using Xpert. Drop-offs were higher among PWH already on ART (Adjusted Odds Ratio [aOR] = 60.65, 95% confidence interval [CI]: 55.11–66.75), and those in multi-month dispensing (MMD; aOR = 1.42, 95% CI: 1.33–1.52) and differentiated models of care (DMoC; aOR = 1.10, 95% CI: 1.03–1.18) versus standard of care. Symptomatic PWH were less likely to experience Xpert drop-offs (aOR = 0.009, 95% CI: 0.008–0.011) than those without symptoms recorded. Of 1746 PWH diagnosed with TB, 6.3% did not initiate treatment, with higher drop-offs in DMoC (aOR = 29.22, 95% CI: 13.29–64.23) and MMD (aOR = 8.65, 95% CI: 2.72–27.48), but lower among symptomatic PWH (aOR = 0.05, 95% CI: 0.03–0.11). Among 1636 who started TB treatment, 25.6% did not complete it. Drop-offs were higher among those with previous TB (aOR = 2.50, 95% CI: 1.71–3.66), and lower among symptomatic PWH (aOR = 0.21, 95% CI: 0.15–0.29).

**Conclusion:**

Findings reveal substantial drop-offs in Xpert testing and TB treatment completion, especially among PWH already on ART. Targeted strategies to identify and retain PWH at highest risk of drop-offs are important for optimising TUTT.

**What this study adds:** This study identifies key determinants of drop-offs across the tuberculosis testing and treatment cascade among people with HIV (PWH) in South Africa. It highlights gaps linked to differentiated HIV care models and asymptomatic PWH, providing actionable evidence to inform targeted interventions and improve integration of tuberculosis services within HIV programmes.

## Introduction

Tuberculosis (TB) remains a leading cause of morbidity and mortality among people with HIV (PWH), despite longstanding global efforts to integrate TB and HIV services.^1,2^ In 2023, the WHO estimated approximately 161 000 HIV-associated TB deaths globally, with sub-Saharan Africa bearing the highest burden.^3^ Given the strong epidemiological overlap between TB and HIV, targeted strategies are needed to improve TB case finding and reduce mortality in this population.^1,3^ The Targeted Universal TB Testing (TUTT) strategy represents a major shift from symptom-based screening to routine rapid molecular testing for TB among all PWH and other high-risk groups, aiming to enhance early TB diagnosis and treatment.^4^ However, the implementation of TUTT remains suboptimal, with multiple points of attrition that limit its impact.^4^ A prerequisite for TUTT implementation is identifying individuals who meet the high-risk criteria for TB testing, after which all eligible PWH should receive annual molecular testing, regardless of symptoms.^4^

The TUTT care cascade consists of sequential steps: molecular testing with Xpert® MTB/RIF Ultra (Cepheid, Sunnyvale, California, United States; hereafter, ‘Xpert’), TB treatment initiation, and treatment completion.^5,6^ While symptom screening remains an important workflow triage tool, the TUTT approach emphasises universal molecular testing for all identified high-risk individuals, regardless of symptoms.^7,8^ Subclinical TB, a form of bacteriologically confirmed disease occurring in individuals without symptoms, is increasingly recognised among PWH and represents a key target population for universal molecular testing.^9^ Following a positive Xpert result, individuals should be promptly initiated on TB treatment, with treatment completion as the final goal to prevent transmission.^8,10^ However, substantial drop-offs occur throughout the TUTT cascade, leading to missed opportunities for timely diagnosis and treatment.^11,12^ In this context, a drop-off refers to a failure to progress from one stage to the next, despite eligibility.^12,13^ These attrition points can compromise the effectiveness of TUTT and hinder TB control among PWH.^12^

Previous studies have identified barriers to TB diagnosis and treatment among PWH, including systemic inefficiencies, limited diagnostic capacity, stigma, and gaps in patient retention.^14,15,16^ However, few studies have examined the specific determinants of drop-offs across the TUTT cascade in high TB/HIV burden settings. Understanding these patterns is essential for designing responsive and targeted interventions.^4^ The national TUTT policy was introduced in 2021, and implementation across provinces and districts was still at an early stage when this study was undertaken. This study therefore aimed to investigate the determinants of drop-offs in the TUTT care cascade among PWH, focusing on three key stages: (1) failure to receive an Xpert test; (2) failure to initiate TB treatment after diagnosis; and (3) failure to complete TB treatment after initiation. By identifying key determinants of drop-offs across these stages, the study provides actionable insights to strengthen TB case finding, improve care continuity, and optimise integration of TB and HIV services.

## Research methods and design

### Study design

We conducted a retrospective analysis of routinely collected patient-level data from healthcare facilities in South Africa, supported by the U.S. President’s Emergency Plan for AIDS Relief (PEPFAR). TB/HIV programme data were extracted from a national electronic register for TB and HIV services. These data are compiled quarterly, verified at the sub-district level, and reported at higher administrative levels. Data verification occurs at facility and sub-district levels through routine validation processes led by information officers, who compare electronic records with source documents, and provide feedback for corrective action. Additional data quality assessments are conducted by Department of Health programme managers, District Support Partners, and PEPFAR staff to ensure accuracy and completeness before reporting. The implementation of TUTT among PWH is integrated into baseline antiretroviral therapy (ART) initiation and ongoing care, with annual Xpert testing recommended for PWH, regardless of symptoms. We analysed data from PWH who accessed HIV care services between October 1, 2021, and September 30, 2022 (PEPFAR fiscal year 2022). A flow diagram illustrating the derivation of the analytic cohorts has been included as Supplementary Figure 1. Data were abstracted between June and July 2024.

### Study population and setting

The study included PWH starting and already on ART in PEPFAR-supported healthcare facilities in the Zululand and eThekwini districts of KwaZulu-Natal province, South Africa. In 2023, HIV prevalence in the province was estimated at 22%, with 1.6 million PWH on ART, 30% of whom were in eThekwini and 8% in Zululand.^17,18^ TB incidence in 2022 was approximately 400 per 100 000 population, and over half of TB notifications were co-infected with HIV.^19,20^ Zululand is predominantly rural, with 75 healthcare facilities and a population of 872 000.^18,21^ eThekwini, encompassing Durban, is urban and the province’s most populous district, with 4.3 million people and 114 healthcare facilities.^18,21^ Data were collected from 21 facilities across both districts: 12 in Zululand (2 community health centres [CHCs], 10 primary healthcare clinics [PHCs]), and 9 in eThekwini (2 CHCs, 7 PHCs). The 21 facilities were selected through stratified random sampling based on facility type and ART patient volume to achieve comparable ART cohort sizes across districts. The study population received care through three ART delivery models: Standard of Care (SoC), which involves monthly clinic visits with a 1-month ART prescription; multi-month dispensing (MMD), where PWH receive 2- or 3-month ART supplies; and differentiated models of care (DMoC), which offer flexible, patient-centred ART collection options, including facility pickups, adherence clubs, support groups, external pickup points, and private pharmacies.^22,23^

### Sampling method and sample size

We selected one rural and one urban PEPFAR-supported district to reflect the heterogeneity of service delivery contexts. Within each district, a stratified random sampling approach was used to select 21 healthcare facilities, based on facility type and ART patient load. The target sample aimed to reflect approximately 50 000 PWH per district. The similar patient totals in rural and urban districts were expected as part of the sampling design.

### Data collection

Data extraction was conducted by the investigator, supported by Department of Health staff and PEPFAR District Support Partners. Programmatic patient-level data line lists were anonymised through the generation of unique study IDs, formed by combining facility identifiers with truncated patient registration numbers. No personal identifiers were collected. The cleaned dataset was stored on a secure, firewall-protected server for analysis. All analyses used routinely collected programmatic TB and ART line list data, which included Xpert results; the dataset was not cross-linked with National Health Laboratory Services databases or electronic medical record systems.

### Data analysis

Data were analysed using Stata version 16 (Stata Corp, College Station, Texas, United States). Descriptive statistics were used to summarise quantitative variables, including frequencies, means, medians, standard deviations (s.d.), interquartile ranges (IQR), proportions, and 95% confidence intervals (95% CI). We conducted univariable and multivariable logistic regression analysis to examine factors associated with drop-off across the three stages of the TUTT cascade. The three outcome variables were defined as follows: (1) Xpert test not done: PWH with no Xpert test documented; (2) Diagnosed with TB but not started on TB treatment: PWH with a positive Xpert result in the study period who did not initiate TB treatment; and (3) Started TB treatment but did not complete it: PWH who initiated TB treatment but did not have a recorded treatment outcome of treatment completion in programmatic data. Invalid or rejected Xpert results and Xpert trace results were excluded from the analysis because downstream eligibility for treatment initiation and completion could not be assessed. Drop-off 2 was defined using programmatic ART outcomes recorded at the time of TB diagnosis, including death, loss to follow-up ≥ 90 days, transfer-out, or no documented TB treatment start. Drop-off 3 was defined using TB programme outcomes recorded during treatment, including death, loss to follow-up, or transfer-out before TB treatment completion. For both drop-off 2 and drop-off 3, death, loss to follow-up, and transfer-out were taken directly from programme outcome fields; no assumptions were made to infer death from loss to follow-up. Deaths before TB treatment initiation were classified under drop-off 2, and deaths during TB treatment were classified as non-completion under drop-off 3. Independent variables were selected a priori based on their reasonable associations with TB testing, initiation, and treatment outcome. These were age, sex, district type, ART delivery model, ART status, WHO four-symptom screen, previous TB history within the preceding 2 years, and viral load testing. All multivariable logistic regression models adjusted for these variables. Results are presented as odds ratios (OR) and adjusted odds ratios (aOR), with 95% CI and *P*-values, with statistical significance *P* < 0.05. Clustering by facility was not applied because facility-level variance was minimal in preliminary diagnostics, and district type and ART delivery model captured major between-facility differences.

### Ethical considerations

Ethical approval was granted by the University of Cape Town, Faculty of Health Sciences, Human Research Ethics Committee (HREC reference number: 037/2023) and the U.S. Centers for Disease Control and Prevention (reference number: 0900f3eb82189915). Approvals were also obtained from the South African National Department of Health and the KwaZulu-Natal Provincial and District Health Departments (reference number: KZ_202302_029). Informed consent was waived because of the use of anonymised, routinely collected data. Data were handled in accordance with national and institutional data protection guidelines.

## Results

### Baseline characteristics

A total of 104 859 PWH were included in the analysis, with a mean age of 37.6 years (s.d. 14.3; IQR 5–71). Children (< 15 years) accounted for 1.9% (*n* = 1991), while adults (≥ 15 years) accounted for 98.1% (*n* = 102 868). Most participants were female (68.7%, *n* = 72 080), and the cohort was evenly distributed between urban (50.8%, *n* = 53 234) and rural (49.2%, *n* = 51 625) districts (Table 1). ART was most accessed through DMoC (60.4%, *n* = 63 343), followed by MMD (28.2%, *n* = 29 613) and SoC (11.4%, *n* = 11 903).

**TABLE 1:** Characteristics of people with HIV in fiscal year 2022† by district type and total.

Variable	Urban (*n* = 53 234)		Rural (*n* = 51 625)		All PWH (*N* = 104 859)	
	*n*	%	*n*	%	*N*	%
**Sex**						
Female	36 100	67.8	35 980	69.7	72 080	68.7
Male	17 134	32.2	15 645	30.3	32 779	31.3
**Age (years)**						
5–14	975	1.8	1016	2.0	1991	1.9
15–24	8782	16.5	8364	16.2	17 146	16.4
25–34	15 800	29.7	15 042	29.1	30 842	29.4
35–44	12 600	23.7	12 302	23.8	24 902	23.7
45–54	6608	12.4	6560	12.7	13 168	12.6
55–64	4334	8.1	4188	8.1	8522	8.1
65+	4135	7.8	4153	8.0	8288	7.9
**ART status**						
Starting ART	4359	8.2	3357	6.5	7716	7.4
Already on ART	48 875	91.8	48 268	93.5	97 143	92.6
**ART delivery model**						
SoC‡	5029	9.4	6874	13.3	11 903	11.4
MMD	17 283	32.5	12 330	23.9	29 613	28.2
DMoC	30 922	58.1	32 421	62.8	63 343	60.4
**W4SS done**						
Yes	53 216	100.0	50 808	98.4	104 024	99.2
No	18	0.0	817	1.6	835	0.8
**W4SS result**						
Positive	2880	5.4	2391	4.6	5271	5.0
Negative	50 336	94.6	48 417	93.8	98 753	94.2
Not applicable	18	0.0	817	1.6	835	0.8
**Previous TB**						
Yes	949	1.8	406	0.8	1355	1.3
No	52 285	98.2	51 219	99.2	103 504	98.7
**Viral load test done**						
Yes	45 181	84.9	42 354	82.0	87 535	83.5
No	8053	15.1	9271	18.0	17 324	16.5

ART, antiretroviral therapy; SoC, standard of care; MMD, multi-month dispensing; DMoC, differentiated models of care; W4SS, WHO Four Symptom Screen; PWH, people with HIV; TB, tuberculosis.

† , fiscal year 2022: 01 October 2021 – 30 September 2022;

‡ , Most individuals in SoC were initiating ART (‘Starting ART’), consistent with clinical practice in which new ART patients attend monthly visits during early treatment.

### Drop-off 1: Xpert test

Among those who received an Xpert test (*n* = 34 932), 5.0% (*n* = 1746) were Xpert-positive. Xpert test drop-offs were substantial, with 66.7% (*n* = 69 927) of PWH not tested. Children aged 5–14 years had the highest drop-offs (66.8%). Among adults, drop-offs ranged from 60.6% to 72.0%, highest in the 55–64 year age group. Drop-offs were similar by sex, but higher among PWH already on ART (71.5%) compared to those starting ART (6.4%). Urban PWH had higher drop-offs (79.5%) than rural PWH (53.5%). By ART delivery model, drop-offs were highest in MMD (75.0%) and DMoC (68.1%) compared to SoC (38.1%). In multivariable analysis, already being on ART was the strongest determinant of not receiving an Xpert test (aOR = 60.65, 95% CI: 55.11–66.75). Having a positive WHO four-symptom screen was associated with lower odds of Xpert test drop-off compared to having a negative symptom screen (aOR = 0.009, 95% CI: 0.008–0.011). People with HIV who were not symptom screened had higher odds of Xpert test drop-off (aOR = 15.56, 95% CI: 11.15–21.72). People with HIV in the rural district were less likely to experience Xpert test drop-off compared to those in the urban district (aOR = 0.17, 95% CI: 0.16–0.18), while those enrolled in differentiated service delivery models were more likely to experience Xpert test drop-off MMD (aOR = 1.42, 95% CI: 1.33–1.52) and DMoC (aOR = 1.10, 95% CI: 1.03–1.18) compared to SoC. Adults were 12% – 13% less likely to drop off than children aged 5–14 years (*P* < 0.05) (Table 2).

**TABLE 2:** Drop-off 1: Factors associated with people with HIV not receiving Xpert test.

Variable	Univariate			Multivariate		
	OR	95% CI	*P*	aOR	95% CI	*P*
**Age (years)**						
5–14 (Ref)	1.00	-	-	1.00	-	-
15–24	0.90	0.81–0.99	0.028	0.87	0.77–0.99	0.040
25–34	0.89	0.81–0.98	0.022	0.88	0.78–1.00	0.046
35–44	0.96	0.87–1.06	0.387	0.89	0.78–1.00	0.059
45–54	1.20	1.09–1.33	< 0.001	0.88	0.77–1.00	0.053
55–64	1.28	1.15–1.42	< 0.001	0.91	0.79–1.03	0.145
65+	1.23	1.11–1.37	< 0.001	0.87	0.77–1.00	0.048
**Sex**						
Male (Ref)	1.00	-	-	1.00	-	-
Female	1.03	1.00–1.06	0.046	0.89	0.86–0.92	< 0.001
**District**						
Urban (Ref)	1.00	-	-	1.00	-	-
Rural	0.30	0.29–0.30	< 0.001	0.17	0.16–0.18	< 0.001
**ART delivery**						
SoC (Ref)	1.00	-	-	1.00	-	-
MMD	4.89	4.67–5.12	< 0.001	1.42	1.33–1.52	< 0.001
DMoC	3.49	3.35–3.63	< 0.001	1.10	1.03–1.18	0.003
**ART status**						
Starting ART (Ref)	1.00	-	-	1.00	-	-
Already on ART	36.79	33.55–40.36	< 0.001	60.65	55.11–66.75	< 0.001
**W4SS result**						
Negative (Ref)	1.00	-	-	1.00	-	-
Positive	0.016	0.014–0.019	< 0.001	0.009	0.008–0.011	< 0.001
Not Screened	9.33	6.71–12.97	< 0.001	15.56	11.15–21.72	< 0.001
**Previous TB**						
No (Ref)	1.00	-	-	1.00	-	-
Yes	0.91	0.81–1.02	0.097	0.68	0.58–0.79	< 0.001
**Viral load done**						
No (Ref)	1.00	-	-	1.00	-	-
Yes	0.83	0.80–0.86	< 0.001	0.79	0.75–0.83	< 0.001

OR, odds ratio; aOR, adjusted odds ratio; CI, confidence interval; Ref, reference group; ART, antiretroviral therapy; W4SS, WHO four symptom screen; TB, tuberculosis; SoC, standard of care; MMD, multi-month dispensing; DMoC, differentiated models of care.

### Drop-off 2: Tuberculosis treatment initiation

Among 1746 PWH diagnosed with TB, 6.3% (*n* = 110) were not initiated on treatment. Of these, 37.3% died before treatment initiation, 24.5% were lost to follow-up from the ART programme (defined as no clinic visit for ≥ 90 days), 23.6% transferred out of the facility while still receiving ART but before TB treatment could be started, and 14.5% had unknown reasons. Since these individuals had not yet started TB treatment, ART programme outcomes were applied to categorise their status at the time of drop-off. In multivariable analysis, treatment drop-off was higher among those in DMoC (aOR = 29.22, 95% CI: 13.29–64.23) and MMD (aOR = 8.65, 95% CI: 2.72–27.48) compared to SoC. A history of TB within the preceding 2 years also increased the odds of drop-off (aOR = 4.46, 95% CI: 2.14–9.28). Conversely, those with a positive WHO four-symptom screen were less likely to drop off (aOR = 0.05, 95% CI: 0.03–0.11), as were PWH already on ART (aOR = 0.43, 95% CI: 0.21–0.88). Viral load testing was associated with a lower likelihood of drop-off (aOR = 0.32, 95% CI: 0.15–0.67). Adults across all age bands had lower odds of drop-off compared to children (*P* < 0.05) (Table 3).

**TABLE 3:** Drop-off 2: Factors associated with eligible people with HIV not started on tuberculosis treatment.

Variable	Univariate			Multivariate		
	OR	95% CI	*P*	aOR	95% CI	*P*
**Age (years)**						
5–14 (Ref)	1.00	-	-	1.00	-	-
15–24	0.26	0.88–0.76	0.014	0.17	0.05–0.60	0.006
25–34	0.32	0.12–0.89	0.029	0.32	0.10–0.98	0.047
35–44	0.28	0.10–0.81	0.018	0.20	0.06–0.65	0.008
45–54	0.26	0.76–0.89	0.032	0.16	0.03–0.70	0.015
55–64	0.54	0.17–1.74	0.303	0.10	0.02–0.44	0.002
65+	0.18	0.04–0.81	0.025	0.18	0.03–1.00	0.051
**Sex**						
Male (Ref)	1.00	-	-	1.00	-	-
Female	1.42	0.96–2.08	0.078	0.76	0.46–1.25	0.280
**District**						
Urban (Ref)	1.00	-	-	1.00	-	-
Rural	1.23	0.83– 1.82	0.298	0.38	0.21–0.68	0.001
**ART delivery**						
SoC (Ref)	1.00	-	-	1.00	-	-
MMD	25.90	10.82–62.04	< 0.001	8.65	2.72–27.48	< 0.001
DMoC	50.44	27.45–92.70	< 0.001	29.22	13.29–64.23	< 0.001
**ART status**						
Starting ART (Ref)	1.00	-	-	1.00	-	-
Already on ART	1.96	1.27– 3.03	0.002	0.43	0.21–0.88	0.020
**W4SS result**						
Negative (Ref)	1.00	-	-	1.00	-	-
Positive	0.05	0.03–0.08	< 0.001	0.05	0.03–0.11	< 0.001
Not Screened	0.47	0.06–3.96	0.488	0.48	0.01–17.59	0.690
**Previous TB**						
No (Ref)	1.00	-	-	1.00	-	-
Yes	1.94	1.12–3.34	0.017	4.46	2.14–9.28	< 0.001
**Viral load done**						
No (Ref)	1.00	-	-	1.00	-	-
Yes	0.56	0.33–0.93	0.026	0.32	0.15–0.67	0.002

OR, odds ratio; aOR, adjusted odds ratio; CI, confidence interval; Ref, reference group; ART, antiretroviral therapy; W4SS, WHO Four Symptom Screen; TB, tuberculosis; SoC, standard of care; MMD, multi-month dispensing; DMoC, differentiated models of care.

### Drop-off 3: Tuberculosis treatment completion

Among the 1636 PWH initiated on TB treatment, 25.6% (*n* = 419) did not complete treatment within the expected 6–9-month period. Of these drop-offs, 71.1% had screened positive for TB symptoms at diagnosis, and the majority were male (55.1% vs 44.9% female). Based on TB programme outcome definitions, the primary reasons for non-completion were loss to follow-up (51.3%), defined as treatment interruption for 2 or more consecutive months; death during the treatment period (32.5%); and transfer-out to another facility before completing treatment (16.2%). PWH with a positive WHO four-symptom screen were less likely to drop-off (aOR = 0.21, 95% CI: 0.15–0.29) compared to those with a negative symptom screen. Conversely, previous TB history increased the likelihood of drop-off (aOR = 2.50, 95% CI: 1.71–3.66). Other variables were not significantly associated with drop-offs in the adjusted model (Table 4).

**TABLE 4:** Drop-off 3: Factors associated with people with HIV not completing tuberculosis treatment.

Variable	Univariate			Multivariate		
	OR	95% CI	*P*	aOR	95% CI	*P*
**Age (years)**						
5–14 (Ref)	1.00	-	-	1.00	-	-
15–24	2.50	0.72–8.62	0.147	2.43	0.61–9.75	0.210
25–34	2.09	0.61–7.13	0.240	2.12	0.53–8.41	0.287
35–44	2.63	0.77–9.00	0.124	2.47	0.62–9.87	0.201
45–54	2.73	0.76–9.75	0.123	2.40	0.58–10.00	0.229
55–64	1.67	0.44–6.27	0.450	1.22	0.28–5.35	0.791
65+	1.89	0.50–7.12	0.348	1.65	0.37–7.38	0.512
**Sex**						
Male (Ref)	1.00	-	-	1.00	-	-
Female	1.04	0.84–1.31	0.703	0.98	0.77–1.24	0.872
**District**						
Urban (Ref)	1.00	-	-	1.00	-	-
Rural	1.12	0.89–1.41	0.315	0.89	0.68–1.16	0.387
**ART delivery**						
SoC (Ref)	1.00	-	-	1.00	-	-
MMD	0.65	0.14–3.01	0.580	0.25	0.06–1.11	0.069
DMoC	1.70	0.67–4.35	0.267	0.63	0.21–1.91	0.418
**ART status**						
Starting ART (Ref)	1.00	-	-	1.00	-	-
Already on ART	1.27	1.01–1.60	0.040	0.92	0.71–1.21	0.568
**W4SS result**						
Negative (Ref)	1.00	-	-	1.00	-	-
Positive	0.27	0.20–0.35	< 0.001	0.21	0.15–0.29	< 0.001
Not Screened	0.20	0.02–1.69	0.138	0.19	0.02–1.71	0.138
**Previous TB**						
No (Ref)	1.00	-	-	1.00	-	-
Yes	2.27	1.59–3.24	< 0.001	2.50	1.71–3.66	< 0.001
**Viral load done**						
No (Ref)	1.00	-	-	1.00	-	-
Yes	0.76	0.54–1.07	0.117	0.77	0.52–1.15	0.210

OR, odds ratio; aOR, adjusted odds ratio; CI, confidence interval; Ref, reference group; ART, antiretroviral therapy; 4SS, WHO Four Symptom Screen; TB, tuberculosis; SoC, standard of care; MMD, multi-month dispensing; DMoC, differentiated models of care.

## Discussion

This study examined drop-offs in the TUTT care cascade among 104 859 PWH, focusing on three key stages: Xpert testing, TB treatment initiation, and completion. The most substantial drop-off occurred at the Xpert testing stage, where two-thirds of PWH (66.7%) were not tested. Xpert test drop-offs were particularly high among PWH already on ART, and in MMD and DMoC. Conversely, drop-offs were lower among symptomatic PWH, female patients, rural PWH, those with a history of TB within the preceding 2 years, and those who had a viral load test done. In this analysis, viral load testing was captured as a binary indicator (test done vs not done), and we interpreted its association with reduced drop-offs primarily as a marker of recent clinical engagement and integrated service delivery, rather than viral load level. Among those diagnosed with TB, 6.3% did not initiate treatment, as a result of death, loss to follow-up, and transfer-out, with drop-offs again highest in DMoC and MMD, and those with previous TB history. At the final stage, one in four PWH (25.6%) who initiated TB treatment did not complete it, with male patients and those with a previous TB history more likely to drop off.

PWH who were asymptomatic or not screened were markedly less likely to be tested, indicating sub-optimal TUTT implementation and continued reliance on symptom-based screening despite universal testing guidance. Similar findings have been reported elsewhere, citing persistent barriers such as healthcare worker shortages, high workloads, limited infrastructure, and poor service integration.^24,25^ A South African study also highlighted provider knowledge and practice gaps, reinforcing the need for targeted training, mentorship, and supervision to strengthen TB testing practices under TUTT.^26,27,28^

Disparities in Xpert test uptake across ART delivery models were striking. Drop-offs were highest among PWH already on ART, particularly those in MMD and DMoC, compared to those in SoC. Although differentiated models aim to improve efficiency and patient-centred care, reduced clinic contact may inadvertently limit TB testing opportunities.^23,29^ Conversely, PWH starting ART, typically managed under SoC, benefit from more frequent visits, which may account for lower drop-off rates.^22^ These findings underscore the need to embed TB testing into differentiated models, particularly at ART refill and review visits, with supporting evidence from India highlighting the value of targeted follow-up for high-risk patients.^22,23,30^

Urban PWH had higher Xpert test drop-offs than their rural counterparts. This finding may reflect urban health system challenges such as overcrowded facilities, decentralised services, and competing programmatic priorities that undermine TB/HIV integration.^31^ Prior studies report similar trends, with lower testing rates despite higher TB positivity in urban areas, where TB incidence can be nearly four times higher than in rural settings.^32^ Our data similarly showed higher drop-offs among male patients in urban settings. While we did not collect data on other urban key populations such as migrants, homeless individuals, and substance users, previous studies have highlighted these groups as being disproportionately affected by TB.^33^ These findings underscore the need for tailored urban interventions and prioritised TB testing during high-volume encounters.^34,35^ Leveraging viral load monitoring visits for bundled TB testing presents a promising strategy to strengthen TUTT implementation, particularly among PWH in MMD and DMoC, who showed lower Xpert drop-offs when viral load testing was performed.^2,36,37^

Our study found that 6.3% of PWH diagnosed with TB did not initiate treatment, with the highest drop-offs observed among those enrolled in MMD and DMoC. This gap underscores key implementation challenges in integrating TB services within differentiated HIV care models. According to clinical guidance, PWH diagnosed with TB while in MMD or DMoC should be recalled and transitioned back to SoC to initiate TB treatment and ensure closer clinical monitoring.^30^ However, reduced clinical contact inherent in MMD and DMoC may hinder timely treatment initiation, particularly when tracking and recall mechanisms are weak.^23^ Additionally, urban PWH were less likely to initiate treatment compared to their rural counterparts, which may reflect fragmented TB/HIV services, lower programmatic prioritisation, or highly mobile patient populations in urban settings.^23^ Notably, TB/HIV service fragmentation persists across many facilities in South Africa, including those in this study, despite efforts toward integration.^11,34^

The most common reasons for non-initiation of TB treatment were death, loss to follow-up, and transfer-out, reflecting both structural and patient-level barriers. Early mortality before treatment initiation is a known risk among PWH with TB, especially when diagnosis is delayed.^38,39^ Studies from Kenya and other high-burden settings have shown that delayed or missed treatment can be fatal, with HIV co-infection significantly increasing mortality risk.^40,41^ Similarly, loss to follow-up is often driven by long travel distances, financial barriers, stigma, and weaknesses in health system follow-up, all of which may prevent patients from returning to initiate TB treatment.^42,43^ Transfer-out, often untracked, disrupts care continuity, particularly in contexts with weak inter-facility communication, and is exacerbated by migration and mobility.^44,45^

The strong association between previous TB history and treatment initiation drop-offs in our analysis may reflect patient fatigue, stigma from repeated TB episodes, or prior negative experiences with the health system.^46^ These findings underscore the importance of psychosocial support and tailored counselling for individuals with a history of TB. Moreover, system-level inefficiencies, such as delays between diagnosis and treatment initiation, prolong infectious periods, increase the risk of transmission, and undermine TB control efforts.^22,47^ To reduce drop-offs, it is essential to strengthen patient tracking systems, facilitate rapid treatment linkage, and integrate TB treatment pathways more explicitly into MMD and DMoC.^7,34,48^ Improving communication between facilities, investing in case managers or linkage officers, and enhancing community-based support could help retain patients through the treatment cascade.^13^ Targeted interventions are also needed in urban areas, where the burden of undiagnosed and untreated TB remains high.^12,33^ Overall, these findings highlight the urgency of closing the treatment initiation gap to improve patient outcomes and interrupt TB transmission.^34^

The 25.6% drop-off in TB treatment completion in our study, with loss to follow-up accounting for more than half of these cases, aligns with findings from numerous studies worldwide.^49,50^ The 32.5% mortality rate among treatment completion drop-offs underscores the persistent threat of TB-related deaths, particularly when exacerbated by co-infections like HIV or the emergence of drug-resistant TB strains.^47,51^ However, this may be an underestimate, as some individuals classified as lost to follow-up may have died, a possibility that could not be verified within our dataset. A history of previous TB also emerged as a determinant of treatment non-completion, consistent with prior studies.^14,52^ Individuals with recurrent TB are more likely to face treatment fatigue, psychosocial challenges, comorbidities, and possible drug resistance, all of which may undermine adherence.^39^ These findings highlight the need for patient-centred strategies that address barriers to treatment completion, such as enhanced adherence counselling, social support mechanisms, and differentiated care models that integrate TB and HIV services more effectively.^53^

Our findings highlight two distinct challenges within the cascade: subclinical TB among asymptomatic PWH, and poor treatment completion among symptomatic individuals. Asymptomatic PWH were more likely to drop off at each stage of the cascade. This aligns with the growing recognition of subclinical TB, a condition prevalent in PWH that sits between latent and active TB, and may progress rapidly to symptomatic disease.^9,54^ Evidence from South Africa suggests that a substantial proportion of bacteriologically confirmed TB cases are asymptomatic.^9,54,55^ While TUTT is instrumental in detecting these individuals, asymptomatic PWH in our study were less likely to initiate or complete TB treatment. This may reflect a lack of perceived illness, diminished urgency to start treatment, and difficulties coping with side effects when transitioning from feeling well to experiencing treatment-related illness.^56^ These findings highlight the need for intensified counselling and tailored treatment options to improve engagement and adherence in this group. Equally concerning, 71% of treatment completion drop-offs occurred among PWH who were symptomatic at TB diagnosis, with most being male patients. Despite clinical signs of illness, this group still disengaged from care, representing missed opportunities for TB transmission reduction and improved outcomes.^48^ These dual challenges highlight the need for differentiated support strategies, including adherence counselling, case management, and active follow-up, for both subclinical and symptomatic PWH.^9,48^

Our study has several limitations. First, findings may not be generalisable beyond the study districts or to other high-risk populations targeted by TUTT because of differences in healthcare infrastructure and programme implementation. However, the insights remain relevant to other high-burden settings facing similar TUTT challenges. Second, as with all routine programmatic data, issues such as incomplete records and potential misclassification may have affected data quality. We mitigated these risks through rigorous quality checks during data collection and analysis. Third, individual-level factors such as socioeconomic status, stigma, or care-seeking behaviours were not assessed and may influence cascade outcomes. Fourth, clinic-level factors such as staffing levels, workflow efficiency, diagnostic capacity, and facility readiness were not available in the programmatic dataset and therefore could not be assessed as potential contributors to cascade drop-offs. Fifth, we excluded Xpert trace results (*n* = 42) from the drop-off analysis because of the absence of clinical follow-up data to assess alignment with the national trace algorithm. Similarly, invalid or rejected Xpert results (*n* = 745) were excluded because these results did not provide a definitive test outcome required to determine progression through the cascade. Sixth, we were also unable to explore associations by viral load level or suppression status, as routinely extracted programmatic data for this analysis did not include individual viral load measurements, and categorised suppression data were not incorporated into the regression models. Seventh, only Xpert results were extracted for this analysis. Drug-susceptible versus drug-resistant TB could not be assessed, as rifampicin resistance information was not consistently available in the programmatic dataset. Lastly, while we observed disparities in Xpert testing, particularly among urban PWH and those in MMD and DMoC, we did not investigate the underlying system-level barriers.

Despite these limitations, the study’s strengths lie in its large sample size and use of real-world programmatic data, which enabled a robust analysis of the TUTT care cascade. The findings identified critical drop-off points and provided novel insights into the performance of ART delivery models and geographical settings. Importantly, the study highlights gaps in TB service integration within HIV differentiated care models, emphasising the need for stronger systems to support TB testing, diagnosis, and treatment among PWH. Future research should explore health system readiness, provider capacity, and integration mechanisms to inform targeted improvements in TUTT implementation. Additionally, future research should evaluate clinic-level determinants of TUTT implementation, including facility capacity, workflow processes, and diagnostic resource availability, to understand how facility-level factors may contribute to drop-offs.

## Conclusion

Our study underscores persistent gaps across the TUTT care cascade among PWH, particularly at the Xpert testing and TB treatment completion stages. Disparities by ART delivery model, geographic setting, and symptom status reveal critical vulnerabilities in the current implementation of TUTT. These findings highlight the need for targeted interventions to improve TB testing and linkage to treatment, particularly among PWH enrolled in differentiated care and those accessing services in urban settings.^27,57^ To improve cascade performance, health systems should prioritise embedding routine Xpert testing into MMD and DMoC workflows, reinforcing patient recall and tracking mechanisms for PWH with confirmed TB, and ensuring rapid transition back to standard care for those requiring closer follow-up.^14,34^ Additionally, interventions that enhance TB/HIV service integration and provide tailored support for individuals with previous TB or subclinical disease may further reduce treatment delays and losses across the care continuum.^35^ Addressing both programmatic and clinical barriers remains essential to realising the full potential of TUTT in reducing TB-related morbidity and mortality among PWH in high-burden settings.^27^
